# Pushing the Boundaries of Psychiatric Neuroimaging to Ground Diagnosis in Biology

**DOI:** 10.1523/ENEURO.0384-19.2019

**Published:** 2019-11-15

**Authors:** Manish Saggar, Lucina Q. Uddin

**Affiliations:** 1 Department of Psychiatry and Behavioral Sciences, Stanford University, Stanford, CA 94305; 2 Department of Psychology, University of Miami, Coral Gables, FL 33124

**Keywords:** computational modeling, diagnosis, neuroimaging, psychiatry

## Abstract

To accurately detect, track progression of, and develop novel treatments for mental illnesses, a diagnostic framework is needed that is grounded in biological features. Here, we present the case for utilizing personalized neuroimaging, computational modeling, standardized computing, and ecologically valid neuroimaging to anchor psychiatric nosology in biology.

## Significance Statement

There is a growing recognition that the boundaries of human neuroimaging data acquisition and analysis must be pushed to ground psychiatric diagnosis in biology. For successful clinical translations, we outline several proposals across the four identified domains of human neuroimaging, namely, (1) reliability of findings; (2) effective clinical translation at the individual subject level; (3) capturing mechanistic insights; and (4) enhancing ecological validity of lab findings. Advances across these domains will be necessary for further progress in psychiatric neuroimaging.

## Introduction

Mental illness affects a large proportion of the global population and has a significant economic impact due to treatment costs and lost productivity. In the United States alone, nearly one in five adults lives with a mental illness (44.7 million in 2016; retrieved from https://www.samhsa.gov/data/). Given this widespread prevalence and societal cost, there is a crucial need for finding ways to prevent and treat mental illness. Despite the challenges of understanding the complexity of the human brain in health and disease, researchers have made large strides in developing tools and methodologies that have allowed us to get a sneak peek into brain functioning over the last two decades. It is, however, shocking that even after such an accelerated pace of discovery in neuroscience and bioengineering, the pace of development for treatment of mental illness has not only been slow but has almost stagnated. This slow progress in the development of psychiatric treatments could be largely attributed to the lack of an accurate and neurobiologically-grounded diagnostic nosology (Redish and Gordon, 2006).

The diagnostic nosology, or disorder classification system, most commonly used in psychiatry [the Diagnostic and Statistical Manual of Mental Disorders (DSM-V)] is built entirely on assessment of symptoms by clinicians. The Research Domain Criteria (RDoC) initiative put forth by the National Institute of Mental Health (Cuthbert and Insel, 2013) aims to address the challenge of revising this diagnostic nosology by creating a framework integrating multiple levels information from genomics to neural circuits and behavior to explore basic dimensions of function across clinical and non-clinical populations. Grounding of a psychiatric diagnosis in biological features can not only potentially provide reliable and valid diagnosis, but can also reveal specific biomarkers to track the course of illness and test the efficacy of new treatments. To borrow an example from Redish and Gordon (2006), Type II diabetes has a complex etiology and pathophysiology; however, unlike psychiatric illnesses, the diagnosis for Type II diabetes is largely based on the biological feature of the amount of hemoglobin A1c in the blood. This biological feature not only helps in precise diagnosis, but also in proper management of blood glucose as well as in testing the efficacy of treatments over time.

Different disciplines of science are actively engaged to illuminate biological features that contribute to major psychiatric illnesses. Specifically, several studies from genetics and neuroimaging are at the frontier. Large-scale genomic investigations have linked molecular genetic variations to major psychiatric illnesses. These studies not only present evidence of heterogeneity and polygenicity of psychiatric disorders, but also reveal that connecting multiple levels of molecular, cellular, and circuit functions to complex human behavior is immensely challenging (Geschwind and Flint, 2015). Further, we are just beginning to understand how observed genetic variants give rise to changes in brain function and behavior (Gandal et al., 2018). Along the same lines, large-scale neuroimaging investigations of both brain structure and function have seemingly failed to pinpoint differences across phenotypically distinct psychiatric diagnosis (Etkin, 2019). Interestingly, instead of finding disorder-specific differences, many large-scale studies have either shown converging evidence toward common circuit dysfunction in brain structure (Goodkind et al., 2015) and/or provided evidence for non-specificity in brain function (Uddin, 2015; Baker et al., 2019).

In this review article, we first outline some of the pan-disciplinary issues that broadly hamper progress in the search for disorder-specific biological features in psychiatry. We then focus specifically on issues unique to neuroimaging research, and lastly present novel avenues that are capitalizing on recent advances in machine learning and biophysical network modeling (BNM) to push the boundaries of personalized neuroimaging.

## Pan-Disciplinary Issues in Anchoring Psychiatric Nosology in Biology

One of the major challenges in finding disorder-specific biomarkers is the fact that clinical symptoms, on which diagnoses are based, may not have a one-to-one mapping to the underlying biological mechanisms. In other words, different biological mechanisms may have led to the same cluster of clinical symptoms. This lack of one-to-one relation between clinical symptoms and biology goes against the traditionally held assumption that symptoms-based stratification could help us discover disorder-specific biomarkers, which in turn would largely explain the observed heterogeneity and comorbidity repeatedly observed in psychiatric disorders (Bijl et al., 1998; Uddin et al., 2017). Thus, several researchers now argue that instead of performing small scale case-controlled studies, where stratification of the sample is based on symptoms, large-scale transdiagnostic/dimensional studies are needed (Etkin, 2019).

The second major and related challenge for biomarker studies is how to validate the observed biotypes. The lack of one-to-one mapping between clinical symptoms and biology postulates that clinical symptoms alone cannot be used to validate the observed biotypes. Thus, newer approaches for validation should be explored, including treatment outcomes and performance on tasks assessing different dimensions of functioning (e.g., RDoC).

The third challenge pertains to lack of group-to-individual translation of findings. Although most studies are conducted at the group level, for the best translational outcomes, such group-level findings need to be reliable even at the single-patient level. However, it has been previously argued that due to the non-ergodicity in human social and psychological processes, the inferences based on group-level data are challenging to generalize to an individual experience or behavior (Fisher et al., 2018). Thus, novel methods that can provide statistically significant as well as behaviorally relevant information at both group and single-participant levels are needed (Saggar et al., 2018).

## Issues Specific to Neuroimaging

To characterize neural substrates of psychiatric disorders, modern neuroimaging tools and sophisticated data analytics have been developed. These neuroimaging methods have already been used to measure a range of neural differences across psychiatric disorders, including, (1) volumetric and morphologic differences (Goodkind et al., 2015); (2) structural connectomics (van den Heuvel et al., 2016); (3) static and dynamic functional connectivity at rest and during task performance (Calhoun et al., 2014); and (4) activation differences across a variety of psychological paradigms (McTeague et al., 2017; Picó-Pérez et al., 2019).

However, neuroimaging as a diagnostic tool still struggles with several specific issues. Here, we broadly classified these issues into four domains: (1) reliability of findings; (2) lack of group-to-individual translation; (3) lack of mechanistic insights; and (4) absence of ecological validity in lab environments and experiments.

The psychological sciences have been reportedly going through a major replication crisis (Open Science Collaboration, 2015; De Boeck and Jeon, 2018), and replicability of previously reported brain-behavior relations in neuroimaging studies are also under heavy scrutiny (Button et al., 2013; Kharabian Masouleh et al., 2019). A variety of explanations have been put forth to account for this lack of replication in neuroimaging findings. First, and perhaps the most straightforward, is the need for large-scale samples (*n* ∼ several hundred of participants) to find reliable and replicable findings (Kharabian Masouleh et al., 2019). Fortunately, there is now an ever-increasing number of consortia that provide such big datasets, including the Human Connectome Project (HCP; Van Essen et al., 2013), UK-Biobank (Smith et al., 2019), and the Adolescent Brain Cognitive Development (ABCD) study (Bjork et al., 2017). Other explanations for the lack of replicability in neuroimaging studies include high corruptibility of data due to head movement and other related artifacts (Power et al., 2012); lack of validation data to show robustness of effects (Davis and Poldrack, 2013); lack of reporting of null findings (Kharabian Masouleh et al., 2019); lack of sharing of raw data or unthresholded statistical parametric maps across labs (Poldrack et al., 2008); and lack of standardized neuroimaging data pre- and post-processing (Eklund et al., 2016).

Although large-scale studies and data sharing are putatively successful in addressing the replicability issue, see a sample list of available datasets at https://sites.google.com/site/publicdatadatabase/, several cautions regarding such studies are worth noting. First, due to larger sample sizes, more focus should be devoted to the clinical significance or effect size as opposed to the statistical significance of a given effect (Etkin, 2019). Second, a large proportion of currently available large-scale data banks are skewed toward “healthy” participants, and not necessarily treatment-seeking participants or patients. Third, most of the available datasets are cross-sectional in nature, as opposed to longitudinal; the assumption being that the effects observed in large-scale cross-sectional studies could putatively translate to individual participants and hence could have clinical significance. However, we also know that the statistical conditions (i.e., ergodicity) needed for such translation to be legitimate are very unlikely to hold in the case of human social and psychological processes (Hamaker, 2012). This raises a major concern for translating insights from studies conducted at the group level to ultimately helping an individual patient in the clinic.

Another, and perhaps deeper issue with neuroimaging-based brain-behavior associations in psychiatric disorders is that even when we find replicable relationships, we are still far from a causal and mechanistic understanding of neural processes underlying psychopathology (Etkin, 2018). Neuroimaging-based brain-behavior associations at best can only provide descriptive information about what is disrupted in the brain due to psychopathology, but not why or how such disruptions occur in the first place (i.e., mechanistic insights).

Lastly, psychiatric neuroimaging, and the field of psychological sciences as a whole, suffers from a lack of connection between highly controlled laboratory environments and the real world. Ecological validity refers to the extent to which research findings can be generalized to real-life settings. In the case of psychiatric neuroimaging, it is unclear how reliably the research findings obtained in a highly controlled laboratory environment with high resolution equipment, sophisticated methods, and typically unmedicated participants could be applicable to real-world clinical care settings with hospital-grade equipment and treatment-seeking patients (Etkin, 2019).

## Pushing the Boundaries of Psychiatric Neuroimaging

**Figure 1.**
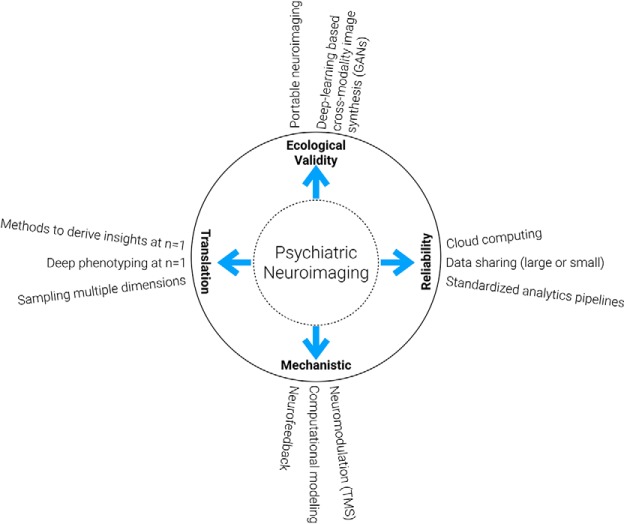
Pushing the boundaries of psychiatric neuroimaging to anchor diagnosis in biological features. Here, we broadly classified the push into four domains: reliability of findings, effective clinical translation, capturing mechanistic insights, and enhancing ecological validity of lab findings.

To address some of the issues with the current state of psychiatric neuroimaging, we provide suggestions for moving the field forward in four main directions (Fig. 1).

### Improving replication and reliability

In the domain of reliability and replication of findings, psychiatry can emulate the momentum that has already developed in the broader fields of neuroimaging and cognitive neuroscience (Poldrack and Gorgolewski, 2014). Specifically, data sharing should be encouraged across labs irrespective of the sample size of the study. It has been previously argued that instead of prioritizing large-scale targeted data collection and sharing (e.g., HCP; Van Essen et al., 2013), it is equally important to also share small-scale data collected by small labs and individual investigators. This approach advocates for “data bazaars” instead of large-scale “data factories” (Poldrack and Gorgolewski, 2014, 2017; Gorgolewski et al., 2017). The Autism Brain Imaging Data Exchange (Di Martino et al., 2014) represents one such grass-roots data sharing initiative. High-impact psychiatric neuroimaging may be accomplished by stitching together data/findings from smaller-scale studies, as large-scale studies may be prohibitively costly. A recent case study by Milham and colleagues provides clear evidence for not only accelerated science, but also massive cost benefits of data reuse as opposed to *de novo* data generation (Milham et al., 2018). Interestingly, during the 2010–2016 period, the cost-benefit analysis of the data shared via the International Neuroimaging Data-sharing Initiative (INDI) consortia alone saved funding agencies north of hundreds of millions of dollars in data collection. Further, availability of such data bazaars could also help with failing faster, developing innovative methods, improving statistical power for future studies (Mumford, 2012), as well as validating older results on newer datasets.

In the area of improving reliability and replicability of findings, there is also a vital need to move away from *ad hoc* data processing workflows toward standardized neuroimaging platforms that provide analysis-agnostic tools with minimal subjective input from the users. Fortunately, several such platforms have been recently developed and are actively curated for most up-to-date processing workflows (Glasser et al., 2013; Esteban et al., 2019). These standardized platforms also produce replicable, transparent and easy-to-use processing workflows, which will in turn ensure the validity of inferences and the interpretability of results. Further, to analyze legacy datasets using newer processing workflows (e.g., surface-base instead of volumetric registration), novel tools are being developed that can integrate legacy datasets without acquisition of accessory sequences (e.g., T2-weighted maps or fieldmaps) with data acquired using state-of-the-art acquisition protocols (Dickie et al., 2019). Lastly, as data sharing grows, large-scale computational frameworks are needed for processing such data. Psychiatric neuroimaging can piggyback on the success of cloud computing for processing such large datasets (or individual data slices) as a web service; thereby collectively accelerating the advancement and rate of breakthroughs (Doel et al., 2017; Madhyastha et al., 2017).

### Improving translational outcomes

For improving translational outcomes, it has been suggested that researchers should not only focus on increasing the sample size but also on collecting more than one (perhaps several) samples from each individual (also known as deep phenotyping or dense scanning). More data from each individual have the obvious advantage to better capture stable traits, supporting the development of personalized medicine (Poldrack et al., 2015; Gratton et al., 2018). However, to robustly study individual differences, and to discern between state-related and trait-related differences, one needs to acquire large quantities (requiring longer scan durations or multiple scan sessions) of artifact-free data (Gordon et al., 2017). Such data collection marathons are even more challenging to acquire when dealing with patients with psychiatric illnesses. In the future, recent advances in the development of software suites that provide scanner operators with head motion analytics in real time (Dosenbach et al., 2017) could be used to acquire artifact-free data. Similarly, to increase participant compliance, especially in pediatric populations, novel protocols that use low cognitive demand stimuli (e.g., abstract movies) that are still somewhat comparable to “resting” state conditions can be used (Vanderwal et al., 2015). Importantly, the use of movie-based paradigms has facilitated the measurement of functional connectivity in awake younger participants (less than seven years; Vanderwal et al., 2019). Although the use of movies as a stimulus (compared with traditional resting state paradigms) increases compliance in terms of arousal and reduced head movement, certain caveats are important to note. First, it is unclear to what degree the transitions in brain activity are associated with extrinsic stimuli (movie watching) versus intrinsic signal fluctuations putatively associated with the wanderings of the mind (during resting state). Second, as most movie paradigms are inherently social in nature, researchers should keep in mind when interpreting results that social processing is invoked (Vanderwal et al., 2019). Third, from the point of view of data aggregation across sites/studies, it is unclear how such aggregation can be possible for different movie paradigms or across movie and resting state paradigms.

**Figure 2.**
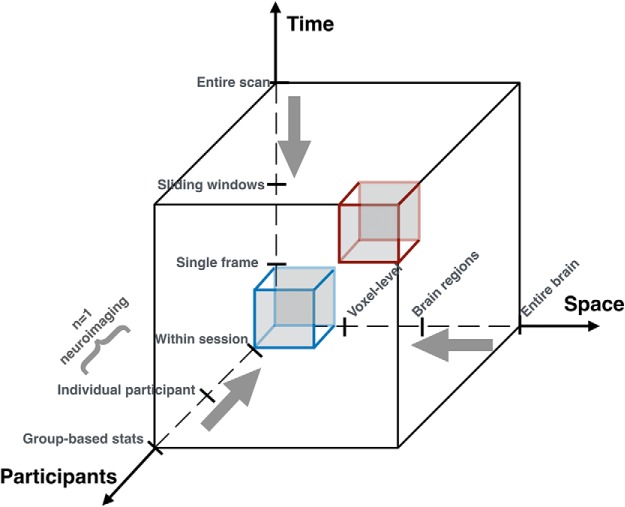
Improving clinical translation of psychiatric neuroimaging findings. Most neuroimaging studies average the data in time, space and across individuals (red cube). In the future, novel analytical methods (e.g., TDA) are needed to examine neuroimaging data at the highest spatiotemporal resolution (blue cube), with the hope that such methods can allow appropriate data-driven resolutions and insights.

For better bench-to-bedside outcomes, it is also important to invest in computational methods that do not require averaging of data (across participants, space or time) at the outset (Fig. 2).

Traditionally, the high spatiotemporal dimensionality and complexity of neuroimaging data has required researchers to reduce the dimensionality of the dataset to increase the signal-to-noise ratio at the cost of potentially useful information. A common example of such reduction in dimensionality across participants is examining group averages. Averaging across the spatial dimension has helped researchers define parcellation schemes and atlases (Schaefer et al., 2018) that are beneficial for developing interventions (e.g., neurofeedback; Sitaram et al., 2017) and increasing interpretability and reproducibility of results across labs. Similarly, averaging across the temporal dimension has been shown to benefit in examination of test-retest reliability of functional connectivity measures. This can also aid in understanding to what degree the functional organization of the brain is stable over time or is state dependent (Gratton et al., 2018). Here, we argue that advances in machine learning and applied mathematics could provide novel avenues to avoid such averaging of data at the outset while distilling complex neuroimaging data into simple, yet vibrant and behaviorally relevant, representations that can be interactively explored to discover new aspects of the data. One such approach was recently developed using topological data analysis (TDA) to generate interactive graphical representations of neuroimaging data at the single participant level (*n* = 1), with a spatiotemporal resolution of individual voxels and time frames to examine whole-brain activity transitions due to intrinsic or extrinsic load (Saggar et al., 2018). One can imagine that such methods could be applied to biologically characterize disorders of attention deficit as those with excessively “rapid” transitions between brain activation patterns, while “inflexibility” or lack of transitions in brain activation could be characteristic of ruminative tendencies such as those observed in depression.

Lastly, for better clinical translation of psychiatric neuroimaging findings, we suggest combining data and insights across multiple units of analysis. Thus, instead of solely relying on measuring the index of functional activity or connectivity associated with a domain or construct, we should focus on also simultaneously assessing well-established measures of physiology (e.g., heart rate, cortisol, etc.), behavior during the task and/or genetic predispositions (Uddin and Karlsgodt, 2018). Such a multivariable approach could not only help reduce the impact of artifacts in neuroimaging data (Power, 2017), but could also provide a more holistic interpretation of findings.

### Providing mechanistic insights

Psychiatric neuroimaging on its own is limited to providing descriptive information about brain-behavior relationships. Thus, even with the best quality neuroimaging data we can only reveal associations that require further testing to confirm cause and effect relationships (e.g., between circuit perturbation and changes in behavior; Etkin, 2018). Although descriptive information is vital, causal mechanistic information could provide the much-needed acceleration in developing treatments or evaluating clinical risk factors. To this end, several techniques can be employed. Here, we briefly discuss four such methods: (1) performing mechanistic comparative trials; (2) designing causal neurostimulation experiments; (3) developing computational models to generate concrete and testable hypotheses for the putative causes of psychopathology; and (4) using neurofeedback to confirm causal links.

Randomized clinical trials are often used to test the efficacy of one treatment over others, while reducing selection and treatment biases (Guyatt et al., 2002). A similar approach, known as comparative mechanistic trials, could also be used to isolate the neural mechanisms perturbed by a specific treatment (Etkin, 2018). In the case of mechanistic trials, randomization could be done across groups to potentially isolate the neural mechanisms (or circuits) through which different interventions influence changes in brain and behavior. Thus, such mechanistic trials could be used to differentiate between two or more possible neural mechanisms underlying a particular psychopathology. Further, as per the principle of clinical equipoise (London, 2017), such comparative mechanistic trials could be considered ethical as the neural mechanisms underlying psychopathology are unknown. Clinical trials, with careful design of comparative treatments, could not only help answer critical questions about the how a particular treatment perturbs neural circuits, but could also reveal factors underlying treatment response (Etkin, 2018).Combining neuroimaging with neuromodulation [e.g., transcranial magnetic stimulation (TMS)] or pharmacology provides another avenue to examine how targeted perturbations affect brain functioning. Such multimodal experimentation could allow for better understanding of why the relation between a certain neuroimaging signal and phenotype of interest was observed. Specifically, it could help answer whether the observed relation is a manifestation of the illness, a compensatory process, or purely an epiphenomenon (Treadway and Leonard, 2016). A recent example of this line of research comes from the work of Brady et al. (2019), where the authors demonstrated that TMS-modulated changes in connectivity between the cerebellum and the right dorsolateral prefrontal cortex causally altered the presence of negative symptom severity in patients with schizophrenia (Brady et al., 2019).

A third potential avenue to provide mechanistic insights associated with psychopathology comes from computational modeling approaches (Friston et al., 2017; Murray et al., 2018). Although a vast area of research, we specifically point out modeling approaches grounded in biology. One such approach is that of large-scale BNMs. A BNM is a system of differential equations describing how the state of each local neuronal population (e.g., firing rate) changes over time in a globally connected network of neuronal populations or brain regions. Here, we focus on biologically realistic BNMs whose parameters are constrained locally for each brain region to be consistent with microscopic properties of neurons (e.g., membrane conductance, time constants of different receptors) and globally for the whole brain by using human connectomes [derived from diffusion-weighted imaging (DWI) data]. The BNMs can be further personalized by fine-tuning model parameters to fit each subject’s own resting state fMRI data (e.g., functional connectivity). Such BNMs are already successfully generating concrete and testable hypothesis regarding neural mechanisms underlying neurologic disorders (Jirsa et al., 2017).

Lastly, another potential avenue for generating mechanistic insights about cognition in general and psychopathology in particular is neurofeedback. Neurofeedback experiments entail providing participants with feedback depicting their brain activity in real time. With practice, participants can learn to modulate this activity on command (Ordikhani-Seyedlar et al., 2016). Although traditionally limited to brain-machine-interfaces to assist individuals overcoming physical disabilities, recent work has shown that neurofeedback could help alleviate symptoms of psychopathology as well (Kim and Birbaumer, 2014; Nicholson et al., 2017). Neurofeedback, being a perturbative approach, could also help in generating mechanistic theories about the brain’s dynamical landscape in health and disease. Further, coupled with the fields of connectomics and network control theory, neurofeedback could become the frontier in understanding human cognition in health and disease (Bassett and Khambhati, 2017).

### Improving ecological validity

In the psychological sciences there has always been a schism between studies which are more naturalistic and hence more ecologically valid versus those conducted in lab environments with greater experimental control. For psychiatric neuroimaging to succeed, biological markers obtained in laboratory environments under ideal conditions need to be equally reliable and applicable to less ideal situations of real-world clinical care (Etkin, 2019). For best bench-to-bedside translation, several factors need to be reconsidered. One of the first factors to reconsider is the cost and availability of high-end neuroimaging devices. MRI is perhaps the most widely used neuroimaging device in the field of psychiatric neuroimaging. Although MRI provides the best spatial resolution for non-invasive human brain imaging, its high cost to acquire and maintain, non-portability, and low ecological validity makes it less than ideal solution for real-world clinical care. Other neuroimaging modalities, like electroencephalogram (EEG) and near-infrared spectroscopy (NIRS) are cost-effective, portable, and more realistic solutions. Thus, future work is needed to translate biological markers derived from MRI to other imaging modalities. One potential venue is to use novel machine learning algorithms [e.g., generative adversarial networks (GANs)] for performing cross-modality image synthesis (Hiasa et al., 2018).

To conclude, there is a growing recognition that the boundaries of human neuroimaging must be pushed to ground psychiatric diagnosis in biology.

## Synthesis

Reviewing Editor: Morgan Barense, University of Toronto Decisions are customarily a result of the Reviewing Editor and the peer reviewers coming together and discussing their recommendations until a consensus is reached. When revisions are invited, a fact-based synthesis statement explaining their decision and outlining what is needed to prepare a revision will be listed below. The following reviewer(s) agreed to reveal their identity: Erin Dickie.

This review gives a useful overview of the current state of the neuropsychiatric imaging field. Overall we found it to be well presented and have only a few minor suggestions/comments.

a) In section 4.1 (Improving reliability and replicability), the authors make mention of the importance of public data-sharing initiatives. I felt that this point could be strengthened even further. Specifically, some of the arguments put forward by Milham et al (2018, Nature Communications), in terms of the economic utility could be used here.

b) The authors state (line 205) that authors should consider using movies instead of resting-state paradigms. While I, personally, don't disagree with the authors on this point. I have had many recent discussions with colleagues who do. So I would not consider the topic of movies vs rest well-understood issue. The authors might consider expanding on this argument with pros and cons.

c) The authors advocate for methods (i.e. topological data analysis) that work at a higher spatiotemporal resolution instead of averaging across dimensions (space, time, participants). For balance, I felt that the authors might want to make some mention of the reasons for averaging. Especially the known benefits of temporal averaging on test-retest reliability of functional connectivity measures, or benefits of some brain parcellations schemes (i.e. well-defined atlases) for increasing the interpretability, and reproducibility, of results.

d) An expanded definition of “comparative mechanistic trials” could be useful. I don't know if your full audience is familiar with that term. Perhaps with an example case of one such trial?

## Author Response

General comment: We would like to thank the editor for giving us an opportunity to revise our manuscript. We greatly appreciate reviewer's suggestions and have accordingly made revisions based on the comments. We hope the manuscript is now suitable for publication. In this document, we provide a detailed response to every comment we received from the reviewer and explain how we addressed their concerns in the revised manuscript.

Comment 1 (Manuscript Instructions): Journal does not allow footnotes. Please remove or incorporate into text of manuscript.

Response: We have removed the footnotes and added the link in the main text.

Comment 2: I think it is important that the significance statement stands on its own, so it would be helpful to state the four domains under consideration.

Response: We thank for this comment and have accordingly added the four domains under consideration to the significance statement.

Comment 3: This review gives a useful overview of the current state of the neuropsychiatric imaging field. Overall, we found it to be well presented and have only a few minor suggestions/comments. In section 4.1 (Improving reliability and replicability), the authors make mention of the importance of public data-sharing initiatives. I felt that this point could be strengthened even further. Specifically, some of the arguments put forward by Milham et al (2018, Nature Communications), in terms of the economic utility could be used here.

Response: We thank the reviewer for bringing recent important work to our attention. We have now added the arguments based on economic utility to section 4.1. The revised text it provided below.

Lines (170-175)

A recent case study by Milham and colleagues provides clear evidence for not only accelerated science, but also massive cost benefits of data reuse as opposed to de novo data generation (Milham et al. 2018). Interestingly, during the 2010-2016 period, the cost-benefit analysis of the data shared via the International Neuroimaging Data-sharing Initiative (INDI) consortia alone saved funding agencies north of hundreds of millions of dollars in data collection.

Comment 4: The authors state (line 205) that authors should consider using movies instead of resting-state paradigms. While I, personally, don't disagree with the authors on this point. I have had many recent discussions with colleagues who do. So, I would not consider the topic of movies vs rest well-understood issue. The authors might consider expanding on this argument with pros and cons.

Response: We agree with the reviewer about providing a balanced argument regarding using movies vs. rest. We have modified the text accordingly to add pros and cons of using low-cognitive demand stimuli (abstract movies) to study large-scale brain networks. The revised text is provided below.

Lines (216-227)

Importantly, the use of movie-based paradigms has facilitated the measurement of functional connectivity in awake younger participants (< 7 years; (Vanderwal, Eilbott, and Castellanos 2019)). Although the use of movies as a stimulus (compared with traditional resting state paradigms) increases compliance in terms of arousal and reduced head movement, certain caveats are important to note. First, it is unclear to what degree the transitions in brain activity are associated with extrinsic stimuli (movie watching) vs intrinsic signal fluctuations putatively associated with the wanderings of the mind (during resting state). Second, as most movie paradigms are inherently social in nature, researchers should keep in mind when interpreting results that social processing is invoked (Vanderwal, Eilbott, and Castellanos 2019). Third, from the point of view of data aggregation across sites/studies, it is unclear how such aggregation can be possible for different movie paradigms or across movie and resting state paradigms.

Comment 5: The authors advocate for methods (i.e. topological data analysis) that work at a higher spatiotemporal resolution instead of averaging across dimensions (space, time, participants). For balance, I felt that the authors might want to make some mention of the reasons for averaging. Especially the known benefits of temporal averaging on test-retest reliability of functional connectivity measures, or benefits of some brain parcellations schemes (i.e. well-defined atlases) for increasing the interpretability, and reproducibility, of results.

Response: We have modified the text to include known benefits of averaging across time and space. The revised text is provided below.

Lines (234-240):

Averaging across the spatial dimension has helped researchers define parcellation schemes and atlases (Schaefer et al. 2018) that are beneficial for developing interventions (e.g., neurofeedback (Sitaram et al. 2017)) and increasing interpretability and reproducibility of results across labs. Similarly, averaging across the temporal dimension has been shown to benefit in examination of test-retest reliability of functional connectivity measures. This can also aid in understanding to what degree the functional organization of the brain is stable over time or is state-dependent (Gratton et al 2018).

Comment 6: An expanded definition of “comparative mechanistic trials” could be useful. I don't know if your full audience is familiar with that term. Perhaps with an example case of one such trial?

Response: We have modified the text to further add clarifications about mechanistic trials and their potential use.

Lines (282-286):

In the case of mechanistic trials, randomization could be done across groups to potentially isolate the neural mechanisms (or circuits) through which different interventions influence changes in brain and behavior. Thus, such mechanistic trials could be used to differentiate between two or more possible neural mechanisms underlying a particular psychopathology.

References:

Milham, Michael P, R Cameron Craddock, Jake J Son, Michael Fleischmann, Jon Clucas, Helen Xu, Bonhwang Koo, et al. 2018. “Assessment of the Impact of Shared Brain Imaging Data on the Scientific Literature..” Nature Communications 9 (1): 2818. doi:10.1038/s41467-018-04976-1.

Schaefer, Alexander, Ru Kong, Evan M Gordon, Timothy O Laumann, Xi-Nian Zuo, Avram J Holmes, Simon B Eickhoff, and B T Thomas Yeo. 2018. “Local-Global Parcellation of the Human Cerebral Cortex From Intrinsic Functional Connectivity MRI..” Cerebral Cortex (New York, N.Y. : 1991) 28 (9): 3095-3114. doi:10.1093/cercor/bhx179.

Sitaram, Ranganatha, Tomas Ros, Luke Stoeckel, Sven Haller, Frank Scharnowski, Jarrod Lewis-Peacock, Nikolaus Weiskopf, et al. 2017. “Closed-Loop Brain Training: the Science of Neurofeedback..” Nature Reviews. Neuroscience 18 (2): 86-100. doi:10.1038/nrn.2016.164.

Vanderwal, Tamara, Jeffrey Eilbott, and F Xavier Castellanos. 2019. “Movies in the Magnet: Naturalistic Paradigms in Developmental Functional Neuroimaging..” Developmental Cognitive Neuroscience 36 (April): 100600. doi:10.1016/j.dcn.2018.10.004.

Gratton, C. et al. Functional Brain Networks Are Dominated by Stable Group and Individual Factors, Not Cognitive or Daily Variation. Neuron 98, 439-452.e5 (2018)
